# A new heterozygous G duplicate in exon1 (c.100dupG) of *gelsolin* gene causes Finnish gelsolin amyloidosis in a Chinese family

**DOI:** 10.1002/brb3.1151

**Published:** 2018-11-12

**Authors:** Xuemin Feng, Hui Zhu, Teng Zhao, Yanbo Hou, Jingyao Liu

**Affiliations:** ^1^ Department of Neurology The First Hospital, Jilin University Jinlin China; ^2^ Department of Internal Medicine The center Hospital of Gongzhuling Jilin China

**Keywords:** Chinese family, duplicate mutation, Finnish gelsolin amyloidosis, gelsolin

## Abstract

**Objectives:**

In this study, we report a case of Finnish gelsolin amyloidosis (FGA) in a Chinese family.

**Methods:**

The proband presented with a range of clinical symptoms that included epileptic seizures and multiple lesions in the brain. Whole exome sequencing of the *Gelsolin* (*GSN*) gene was performed, and the GSN mutation was identified through comparison with the known human genome sequences using Genetic Testing Intelligent Execution System.

**Results:**

The *GSN* gene sequencing revealed that a heterozygous G duplicate in exon1 (c.100dupG) of the *GSN* gene, which caused a frameshift in GSN transcript translation in the proband, his mother and daughter, but his brother did not have it.

**Conclusion:**

We presented a new autosomal dominant heterozygous G duplicate mutation in exon1 of *GSN* gene, leading to FGA in a Chinese family.

## 1 INTRODUCTION

Finnish gelsolin amyloidosis (AGel amyloidosis, FGA) is one of the most common Finnish heritage diseases (Kiuru‐Enari & Haltia, 2013). FGA was first described in 1969 by a Finnish ophthalmologist, Jouko Meretoja (Meretoja, 1969). The number of FGA patients in Finland is estimated to be approximately 400–1,000. Although it has been mostly found in Finland or in patients of Finnish descent (Kiuru‐Enari & Haltia, 2013), it has also been identified in Japan (Taira et al, 2012), America (Kiuru, 1998), India (Maramattom & Chickabasaviah, 2013), and Korea (Park et al, 2016). The disease most likely remains underdiagnosed or even misdiagnosed worldwide (Juusela et al, 2009).

Finnish gelsolin amyloidosis is caused by a mutation in the gene coding for gelsolin (GSN), which is located on chromosome 9 (Kwiatkowski, Westbrook, Bruns, & Morton, 1988). It is autosomal dominantly inherited and has a penetrance of 100% (Kiuru‐Enari & Haltia, 2013). Furthermore, it was initially found that the disease was caused by a point mutation c.640G‐A in the gene coding for GSN. However, another less common point mutation, c.640G‐T, was reported at a later date (Kiuru‐Enari & Haltia, 2013). These point mutations cause defective folding of GSN protein, which makes the protein accumulate as GSN amyloid in various tissues. GSN mutations have been found in all molecular genetically characterized FGA patients in Finland (Nikoskinen, Schmidt, Strbian, Kiuru‐Enari, & Atula, 2015).

The symptoms of FGA are multifaceted and have been considered to depend on the tissues in which the GSN amyloid accumulates. The most common symptoms include cranial neuropathies, mild peripheral neuropathy, carpal tunnel syndrome, lattice corneal dystrophy type II (LCD‐2), glaucoma/cataract, and cutis laxa. For most of these patients, lattice corneal dystrophy (LCD) has an early onset and can be used as an indicator to diagnose the disease at an earlier period. Polyneuropathy (Kiuru‐Enar, Somer, Seppäläinen, Notkola, & Haltia, 2002), facial nerve paresis, and impaired hearing are common symptoms, while dysarthria is not very common, but noteworthy in FGA (Kiuru, 1992). FGA typically develops quite slowly at different paces in different patients, which usually takes several years or even decades (Atula et al, 2016).

In the present study, we report a case of FGA with a frameshift mutation in exon 1 of GSN in a Chinese family.

## 2 SUBJECTS AND METHODS

### 2.1 Subjects

The present study was approved by the local Ethics Committee of Jilin University, Changchun, China. Four people from a Chinese family were studied after a signed written informed consent was provided by the participants. These participants were the proband, the proband's mother, the proband's daughter, and the proband's brother.

### 2.2 Methods

#### 2.2.1 Computed tomography and MRI

The proband's brain and his family members’ brains were examined by standard computed tomography (CT) and magnetic resonance imaging (MRI) imaging.

#### 2.2.2 Identification of GSN mutation

Blood samples were obtained from the patients in the family, and the DNA was isolated. Whole exome sequencing was performed on Illumina HiSeq 2500 (Illumina, San Diego, CA). The GSN mutation was identified through comparison with the known human genome sequences using Genetic Testing Intelligent Execution System (PrMat, Shanghai, China).

## 3 RESULTS

### 3.1 Clinical manifestations

**Figure 1:**
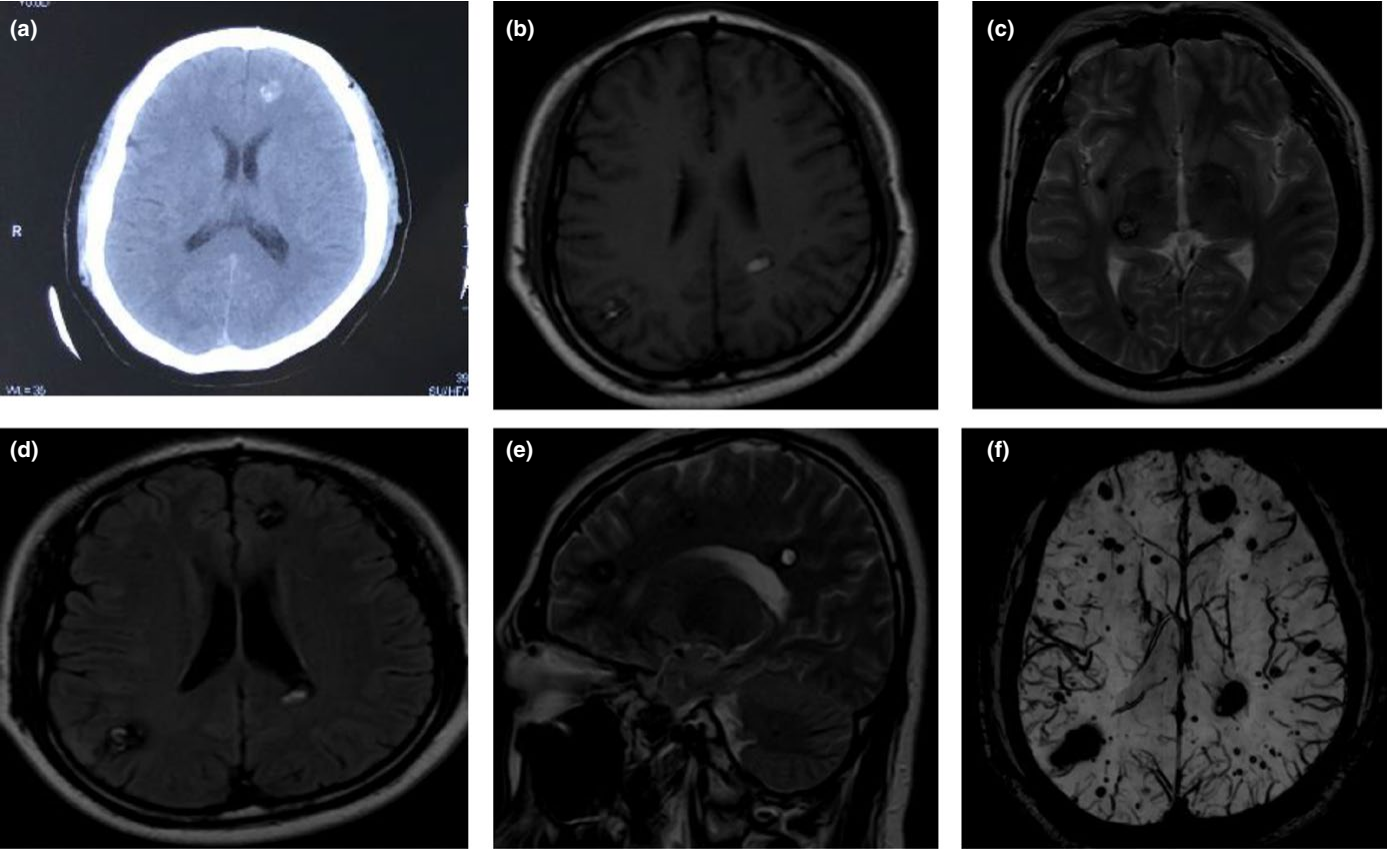
The brain CT and MRI images of the proband's brain is shown. Panel a: CT of the proband. Panel b–e: axial T1, T2‐weighted MRI of the proband's brain; an irregular mixed‐density lesion is shown in the cerebral hemisphere; the hyperdense region represents calcification with bleeding. Panel f: SWI revealed multiple hypointense signals in the cerebral hemisphere of the brain

The proband was a 39‐year‐old male who presented with seizures three times within three days in China. There were no symptoms presenting in the eyes, peripheral nerves, and skin. CT revealed a hemorrhagic lesion in the bilateral basal ganglia (Figure 1a) of his brain. The MRI (Figure 1b–e) revealed multiple cerebral cavernous malformation lesions in the cerebral hemisphere of the brain. T2‐weighted images revealed that the lesions were typically characterized by an area of various signal intensities. Susceptibility‐weighted imaging (SWI) (Figure 1f) revealed multiple hypointense areas in the cerebral hemisphere of the brain.

His 69‐year‐old mother had no neurologic symptoms. However, the MRI also revealed multiple cerebral cavernous malformations in the cerebral hemisphere and cerebellum of her brain. The proband's brother and daughter had no neurologic symptoms, no history of neurologic disorders, and normal neurological examination results. At the same time, the MRI did not reveal any lesions in the cerebral hemisphere of their brains.

### 3.2 Genetic analysis

**Figure 2:**
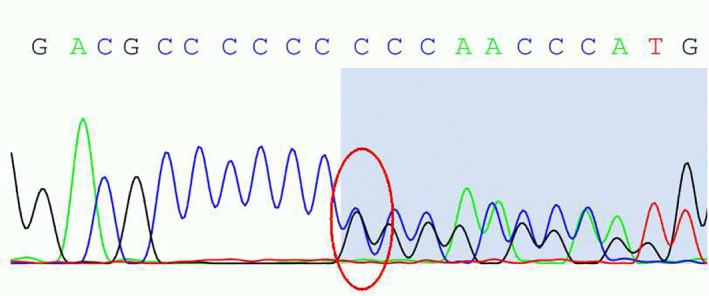
Sequencing of the *GSN* gene DNA from a member of the patient's family. The graph shows the heterozygous G duplicate in exon1 (c.100dupG) of the *GSN* gene

**Figure 3:**
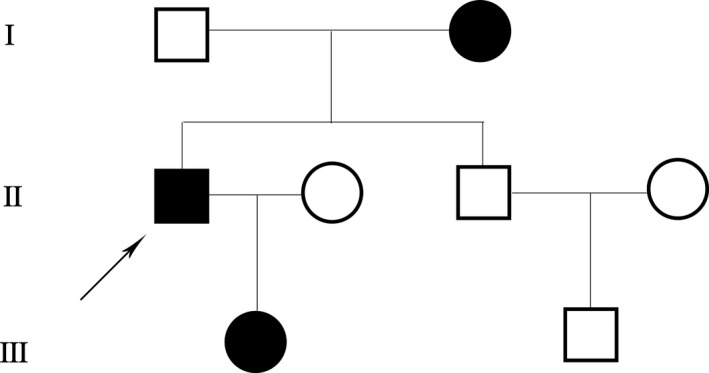
Pedigree of a family with Finnish gelsolin amyloidosis. □, male; ■, male patient; ○, female; ●, female patient; ↗, proband

The family members’ DNA sequences, which contained the coding exons and intron–exon boundaries of GSN were sequenced (Figure 2) and screened. A heterozygous G duplicate in exon1 (c.100dupG) of the *GSN* gene was found in the *GSN* gene of the proband, his mother, and his daughter (Figure 2). This heterozygous duplicate was predicted to cause a reading frameshift when the mutant GSN transcript is translated and change the sequence of the amino acids in gelsolin protein p.(Ala34fs) (shift code mutation). Based on the clinical manifestations and genotypic analysis results, a pedigree of the family with FGA malformation was constructed (Figure 3).

## 4 DISCUSSION

In the present study, a new reading frameshift mutation was identified in the *GSN* gene, which revealed an autosomal dominant inheritance pattern, and led to FGA in a Chinese family.

Clinical manifestations including seizures, multiple cerebral cavernous malformation, and hemorrhagic lesions in the proband's brain, and multiple cerebral cavernous malformation lesions in his mother's brain, complied with most of the common symptoms of FGA, suggesting that the patients might have FGA (Kiuru‐Enar et al., 2002). The presence of the heterozygous G duplicate in exon1 (c.100dupG) of the *GSN* gene further supports that the disease of these patients was GSN mutation‐mediated FGA (Kiuru‐Enar et al., 2002; Kwiatkowski et al., 1988; Nikoskinen et al, 2015). The presence of these clinical manifestations and the mutation in the *GSN* gene in the proband and his mother, and the presence of the mutation in GSN without any clinical manifestation in the proband's daughter, support the observations that FGA develops at different paces in different patients (Atula, 2016). The presence of seizures and hemorrhagic lesions in the proband and the absence of seizures or hemorrhagic lesions in his mother and daughter also implicate that there may be a sex difference in response to the mutation in the *GSN* gene (Atula, 2016).

The mutation found in the present study was a heterozygous G duplicate in exon1 (c.100dupG) of the *GSN* gene, which was different from previously identified *GSN* gene mutations, such as the G to A transition at nucleotide 654 (Maury, Kere, Tolvanen, & de la Chapelle, 1990) and the G to T transversion at nucleotide 654 (de la Chapelle et al., 1992), indicating that the mutation had not been identified before. Since the proband's brother was normal and had no mutations, the GSN detected in the proband's mother must be heterozygous for the identified mutation. Although the proband and his daughter could not be excluded to be homozygous for the identified mutation based on the pedigree, the *GSN* gene sequencing result demonstrated that they were heterozygous for the mutation. Combined with the dominant phenotypical expression of the clinical manifestations in the proband and his mother, it is obvious that the mutation is an autosomal dominant mutation, which is in agreement with the observations for other identified mutations in the *GSN* gene (Kiuru‐Enari & Haltia, 2013).

The heterozygous duplicate found in the present study is predicted to cause a reading frameshift when the mutant GSN transcript is translated, and change the sequence of amino acids in gelsolin protein. The change in the sequence of amino acids in gelsolin protein may cause defective folding of gelsolin protein, which may induce the protein to accumulate as gelsolin amyloid in the patient's brain tissues (Kiuru‐Enar et al., 2002; Nikoskinen et al, 2015), damage the structures of the brain tissues, and lead to these manifestations in the patients. However, this proposed mechanism should be further elucidated through experiments.

In conclusion, a new duplicate mutation, which causes a reading frameshift in the *GSN* gene, was identified. This revealed an autosomal dominant inheritance pattern, which led to FGA in a Chinese family.

## CONFLICT OF INTEREST

There are no conflict of interests.

## AUTHOR CONTRIBUTIONS

Xuemin Feng and Hui Zhu carried out the molecular genetic studies, participated in the sequence alignment, and drafted the manuscript. Yanbo Hou participated in the sequence alignment. Teng Zhao participated in the design of the study. Jingyao Liu conceived of the study, participated in its design and coordination, and helped to draft the manuscript. Xuemin Feng wrote the paper. All authors read and approved the final manuscript.
